# Detection of *Mycobacterium tuberculosis* in extrapulmonary biopsy samples using PCR targeting IS*6110*, *rpoB*, and nested-*rpoB* PCR Cloning

**DOI:** 10.3389/fmicb.2015.00675

**Published:** 2015-07-03

**Authors:** Hossein Meghdadi, Azar D. Khosravi, Ata A. Ghadiri, Amir H. Sina, Ameneh Alami

**Affiliations:** ^1^Department of Microbiology, School of Medicine, Ahvaz Jundishapur University of Medical Sciences, Ahvaz, Iran; ^2^Health Research Institute, Infectious and Tropical Diseases Research Center, Ahvaz Jundishapur University of Medical Sciences, Ahvaz, Iran; ^3^Department of Immunology, School of Medicine, Ahvaz Jundishapur University of Medical Sciences, Ahvaz, Iran; ^4^Cell and Molecular Research Center, Ahvaz Jundishapur University of Medical Sciences, Ahvaz, Iran; ^5^Danesh Medical Laboratory, Ahvaz, Iran

**Keywords:** *Mycobacterium tuberculosis*, extrapulmonary tuberculosis, polymerase chain reaction, biopsy, TA cloning

## Abstract

Present study was aimed to examine the diagnostic utility of polymerase chain reaction (PCR) and nested PCR techniques for the detection of Mycobacterium tuberculosis (MTB) DNA in samples from patients with extra pulmonary tuberculosis (EPTB). In total 80 formalin-fixed, paraffin-embedded (FFPE) samples comprising 70 samples with definite diagnosis of EPTB and 10 samples from known non- EPTB on the basis of histopathology examination, were included in the study. PCR amplification targeting IS*6110*, *rpoB* gene and nested PCR targeting the *rpoB* gene were performed on the extracted DNAs from 80 FFPE samples. The strong positive samples were directly sequenced. For negative samples and those with weak band in nested-*rpoB* PCR, TA cloning was performed by cloning the products into the plasmid vector with subsequent sequencing. The 95% confidence intervals (CI) for the estimates of sensitivity and specificity were calculated for each method. Fourteen (20%), 34 (48.6%), and 60 (85.7%) of the 70 positive samples confirmed by histopathology, were positive by *rpoB*-PCR, IS*6110*-PCR, and nested-*rpoB* PCR, respectively. By performing TA cloning on samples that yielded weak (*n* = 8) or negative results (*n* = 10) in the PCR methods, we were able to improve their quality for later sequencing. All samples with weak band and 7 out of 10 negative samples, showed strong positive results after cloning. So nested-*rpoB* PCR cloning revealed positivity in 67 out of 70 confirmed samples (95.7%). The sensitivity of these combination methods was calculated as 95.7% in comparison with histopathology examination. The CI for sensitivity of the PCR methods were calculated as 11.39–31.27% for *rpoB*-PCR, 36.44–60.83% for IS*6110*- PCR, 75.29–92.93% for nested-*rpoB* PCR, and 87.98–99.11% for nested-*rpoB* PCR cloning. The 10 true EPTB negative samples by histopathology, were negative by all tested methods including cloning and were used to calculate the specificity of the applied methods. The CI for 100% specificity of each PCR method were calculated as 69.15–100%. Our results indicated that nested-*rpoB* PCR combined with TA cloning and sequencing is a preferred method for the detection of MTB DNA in EPTB samples with high sensitivity and specificity which confirm the histopathology results.

## Introduction

Despite the great advances in diagnosis and treatment of tuberculosis (TB), *Mycobacterium tuberculosis* (MTB) is still regarded as a major public health concern (Nahid et al., 2006; Tsara et al., 2009). World Health Organization (WHO), reported that nearly one-third of the global population infected with a member of the MTB complex (MTBC). Approximately 8.6 million new cases and 1.3 million deaths occur annually (World Health Organization, 2013). In most cases, TB affects the lungs, but the disease can potentially influence all organs of the body. In low incidence countries, approximately 15% of the cases are Extrapulmonary TB (EPTB; Hillemann et al., 2011). Conventional detection and identification of MTB, based on combination of microscopic examination and culture has been accepted as the gold standard, however, culture method despite being sensitive, is time-consuming and can takes several weeks (Moure et al., 2011), and direct microscopy and Ziehl-Neelsen staining do not provide sufficient sensitivities (about 20–80%) (Cattamanchi et al., 2010). EP specimens usually contain small number of bacteria, consequently culture and staining for acid-fast bacilli associated with the least sensitivity (Chakravorty et al., 2005). The histological examination of tissue samples is the traditional technique for diagnosis of EPTB which reveals the presence of granulomatous inflammation and caseous necrosis (Almadi et al., 2009). However, this method does not distinguish between EPTB and infections caused by other granulomatous diseases such as nontuberculous mycobacterial (NTM) diseases, sarcoidosis, leprosy and systemic lupus erythematosus (Mehta et al., 2012). The polymerase chain reaction (PCR) technique is a useful tool for rapid diagnosis of MTB in EP samples with high sensitivity and specificity (Supiyaphun et al., 2010). Among EP specimens, formalin-fixed, paraffin-embedded (FFPE) blocks can be used to study the tubercular infections. These samples cannot be cultured and may be unsuitable for PCR, because chemical alterations of the DNA (as degradation of the DNA) reduces the sensitivity of the PCR, but it can be improved by changing the amplification parameters such as using various targets with different sizes (Barcelos et al., 2008). The PCR can be used for a variety of targets such as IS*6110*, *devR*, *mtp*40, 16S rRNA, *rpoB* gene, etc (Balamurugan et al., 2006; Osores et al., 2006; Yang et al., 2011). In most MTB strains, there are 10 to 16 copies of IS*6110* repetitive element (Piersimoni and Scarparo, 2003), though in some strains, particularly strains from Southeast Asia, there is no copy of this sequence or their number is negligible (Lok et al., 2002). So in such strains, the *rpoB* gene can be used as a target for identification of MTBC. *rpoB* encodes the B subunit of RNA polymerase, the *rpoB* nucleotide sequences comprising the Rif^r^ region, which is associated with resistance to rifampin (Nisha et al., 2012). Previously Yun et al. (2005) used nested *rpoB*-PCR combined with TA cloning to improve the detection of MTB in joint biopsy sections successfully.

In the present study, The PCR technique targeting the *rpoB* gene and IS*6110* fragment and also nested PCR targeting *rpoB* gene were applied to formalin fixed, paraffin-embedded tissue samples from patients suspected of having EPTB. Because of very low number of bacteria in such samples which makes PCR bands usually weak or undetectable by agarose gel electrophoresis, TA cloning-sequencing was used for PCR confirmation.

## Materials and Methods

### Clinical Specimens Preparation

Eighty FFPE samples were collected from the departments of Pathology, university teaching hospitals, Ahvaz Jundishapur University of Medical Sciences, Iran. The initial proposal of the work was approved in the University high research and ethics combined committee. Seventy samples were belonged to patients with definite diagnosis of EPTB made on the basis of histopathology examination of FFPE tissues as gold standard method showing necrotizing granulomatous, to assess the sensitivity of PCR analyses, and ten samples were from known non-EPTB samples as true negatives to estimate the specificity of PCR technique. The samples type were: lymph node 29, pleural effusion 22, skin 8, breast 6, colon 4, thyroid 4, soft tissue 4, bladder, omentum, and kidney each one sample. The samples belonged to 36 male (45%) and 44 female (55%) patients. From each block, 2–3 sections of 8–10 μm with an average surface area of 1 cm^2^ were prepared for DNA extraction.

### DNA Extraction and PCR Amplification

QIAamp DNA FFPE Tissue kit (Qiagen, Germany) was used for DNA extraction according to the manufacturer’s instruction. Two different target genes were used in PCR technique.

PCR amplification for *rpoB* gene was performed by formerly reported set of primers [MF, 5′-CGACCACTTCGGCAACCG-3′ and MR, 5′-TCGATCGGGCACATCCGG-3′] (Kim et al., 1999), which amplify a fragment of 342-base pair (bp) of the target gene. DNA amplification was performed in a thermocycler nexus gradient (Eppendorf, Germany), in a final volume of 25 μl containing 10x PCR buffer, 1.5 mM Mg Cl_2_, 10 mM dNTPs, 0.5 μM of each primer, 1.5 U super *Taq* polymerase and 5 μl of template DNA. All the reagents were purchased from Roche Company, Germany. The amplification program was consisted of initial denaturation at 95° C for 5 min, followed by 35 cycles of denaturation at 94° C for 30 s, annealing at 60° C for 30 s, extension at 72° C for 45 s, and a final extension at 72° C for 5 min.

PCR amplification for IS*6110* fragment was performed by a set of primer [MTB1, 5′-CCTGCGAGCGTAGGCGTCGG-3′ and MTB2, 5′-CTCGTCCAGCGCCGCTTCGG-3′] (Eisenach et al., 1990). The primers are specific for MTBC and amplify a 123-bp fragment of repetitive sequence of IS*6110* gene. The reaction mixture was the same as prepared for *rpoB* gene and the amplification condition consisted of initial denaturation at 95° C for 4 min, followed by 35 cycles of denaturation at 94° C for 1 min, annealing at 68° C for 2 min, extension at 72° C for 1 min and final extension at 72° C for 7 min. *M. tuberculosis* H37Rv standard strain as positive control and a non-necrotizing granulomatous inflammation FFPE tissue sample and also master mix without DNA sample as two negative controls were included in each PCR run.

### Nested rpoB PCR

A nested PCR based on the amplification of the *rpoB* gene of MTB was performed later for samples with inadequate DNA and showed weak bands on the first PCR amplification. The first round PCR was done using the formerly reported outer primers of TB1, 5′-ACGTGGAGGCGATCACACCGCAGACGT-3′, and TB2, 5′-TGCACGTCGCGGACCTCCAGCCCGGCA-3′, which amplify a region of 205 bp of target gene (Kim et al., 2001). The second round (Nested) PCR used the amplicon of the first round as template and the reported inner primers of TR9, 5′-TCGCCGCGATCAAGGAGT-3′, and TR8, 5′-TGCACGTCGCGGACCTCCA-3′, which amplify a 157 bp fragment (Cheng et al., 2007).

The total reaction volume in the first PCR round was 25 μl and contained 0.2 μM of each dNTP, 1.5 mM MgCl_2_, 0.5 μM of each primers, 1.5 U of Super Taq^TM^ DNA polymerase (Roche, Germany), 14 μl sterile distilled water and 5 μl of Template DNA. The PCR conditions consisted of initial denaturation at 95° C for 4 min, followed by 30 cycles of denaturation at 94° C for 30s, annealing at 70° C for 30 s, extension at 72° C for 30 s and final extension at 78° C for 5 min. For nested PCR, 2 μl of the first round amplicon was diluted (200-fold) and transferred to 48 μl of a master mix solution (total reaction was 50 μl) containing the same concentrations as described above, except that 36.6 μl of sterile distilled water was used. The PCR conditions consisted of initial denaturation at 94° C for 5 min, followed by 28 cycles of denaturation at 94° C for 30 s, annealing at 55° C for 30 s, extension at 72° C for 30 s and final extension at 72° C for 5 min.

The products of each PCR assay were analyzed by gel electrophoresis on 3% agarose (w/vol.) containing 1 μg/ml ethidium bromide (Fisher Scientific, USA). Results were recorded using a gel documentation apparatus (AlphaImager Systems, Protein Simple, USA). A 50 bp DNA ladder was used as size marker (Roche, Germany). The PCR products were sequenced for further analysis. SPSS software (SPSS Inc no. 13) was used for data analysis. Receiver-operating-characteristic curves (ROC) were calculated and expressed as areas under the curve, with an asymptotic 95% confidence interval (CI).

### Thymine-Adenine (TA) Cloning

The samples yielding a weak positive results in the nested-*rpoB* PCR, and all negative samples (including the 10 EPTB true negatives by histopathology examination), were subjected to cloning using a TA cloning kit (Invitrogen, USA), according to the supplier instructions. In brief, the ligation reaction was prepared and was transformed into competent *Escherichia coli* TOP10 cells. Recombinant pCR® 2.1: *rpoB* clones. The cloning process was confirmed by application a PCR using inner primers (TR9 and TR8) after a DNA extraction step based on simple boiling method. Simultaneously, a few transformed colonies were cultured in Luria-Bertani medium containing ampicillin. The plasmid DNA was then extracted by use of The GF-1 Plasmid DNA Extraction Kit (Vivantis, Malaysia).

The 95% CI for the estimates of sensitivity and specificity were calculated for each method used according to Clopper-Pearson (Agresti, 2002).

### Nucleotide Sequencing and Sequence Analyses

The nucleotide sequence of the *rpoB* PCR product (157 bp) was determined by using inner primers (TR9 and TR8) and The nucleotide sequences of the purified plasmid by using universal M13 forward and reverse primers which were supplied in the TA cloning kit. Sequences were aligned by using blast to the reference DNA sequence.

## Results

**FIGURE 1:**
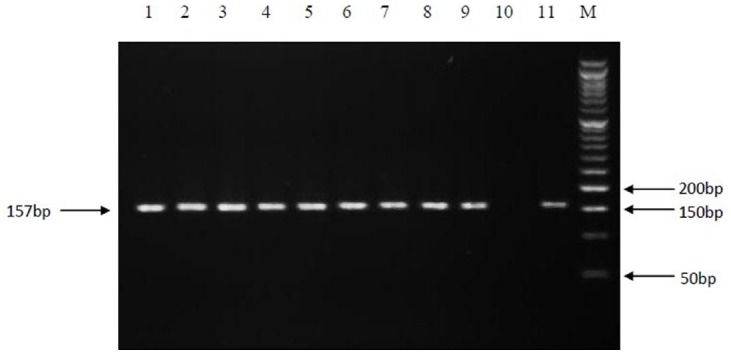
**PCR amplification of the 157 bp fragment of *rpoB* gene.** Electrophoresis of PCR products on a 3% agarose gel. Lanes: 1 to 9, positive clinical isolates; 10, negative control; 11, positive control (*M. tuberculosis* H37Rv); M, Molecular size marker.

From the total 70 samples confirmed as EPTB positive according to the histopathology examination (gold-standard), 14 samples (20%) were positive by PCR amplification of the *rpoB* gene, while by IS*6110*- based amplification, 34 samples (48.6%) and by nested-*rpoB* PCR, 60 samples (85.7%) were positive for the presence of MTBC (Figure 1).

**FIGURE 2:**
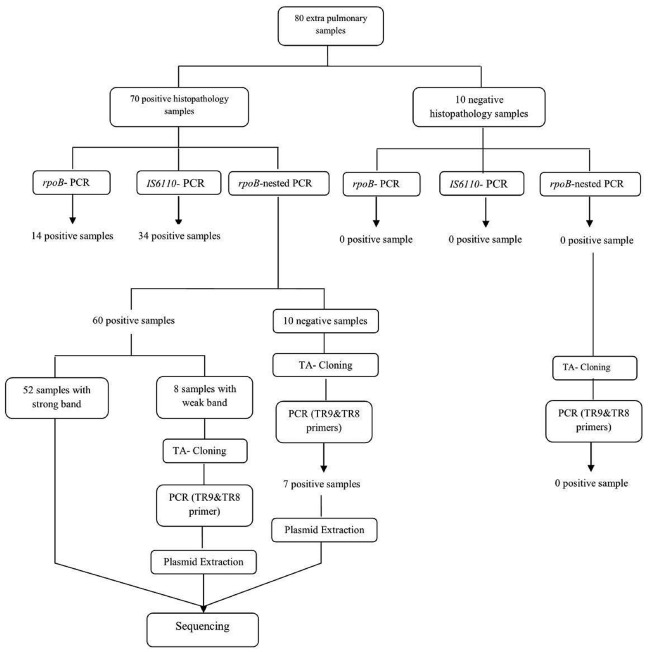
**Schematic scheme of processing of samples with PCR methods and cloning**.

By performing TA cloning on samples that yielded weak (*n* = 8) or negative results (*n* = 10) in the PCR methods, we were able to improve their quality for later sequencing. All samples with weak band and 7 out of 10 negative samples, showed strong positive results after cloning. So nested-*rpoB* PCR cloning revealed positivity in 67 out of 70 confirmed samples (95.7%) (Figure 2). The sensitivity of these combination methods was calculated as 95.7% in comparison with histopathology examination. The sequences of cloned plasmid were compared with MTB H37Rv (Genbank), which 98–100% homology was revealed. In Table 1, the distribution of positive samples according to their origin by application of each PCR method is presented.

**TABLE 1:** **Distribution of positive samples according to PCR amplification methods used**.

**Clinical sample (No.)**	***rpoB*-based PCR No. (%)**	***IS6110*-based PCR No. (%)**	***rpoB*-nested PCR No. (%)**	***rpoB*-nested PCR with Cloning, No. (%)**
Lymph node (25)	10 (40)	15 (60)	22 (88)	23 (92)
Pleural effusion (19)	4 (21)	10 (52.6)	17 (89.5)	18 (94.7)
Skin (8)	–	4 (50)	6 (75)	8 (100)
Breast (5)	–	2 (40)	4 (80)	5 (100)
Colon (4)	–	1 (25)	4 (100)	4 (100)
Soft tissue (4)	–	1 (25)	3 (75)	4 (100)
Thyroid (2)	–	–	2 (100)	2 (100)
Kidney (1)	–	1 (100)	1 (100)	1 (100)
Bladder (1)	–	–	1 (100)	1 (100)
Omentum (1)	–	–	–	1 (100)
Total (70)	14 (20)	34 (48.6)	60 (85.7)	67 (95.7)

The details of samples underwent cloning are presented in Table 2. In certain clinical samples i.e. skin, colon, soft tissue, TA cloning showed twice positivity rate compared to the *rpoB*-nested PCR alone. Three samples (two lymph nodes and one pleural effusion) were negative by nested- *rpoB* PCR with cloning.

**TABLE 2:** **The EPTB histopathologic-confirmed samples with weak positive results or negative by PCR methods candidate for TA cloning**.

**Clinical sample that were cloned (No.)**	**Weak positive positive**	**Undetectable by agarose gel electrophoresis**
Lymph node (5)	2	3
Pleural effusion (2)	1	1
Skin (4)	2	2
Breast (2)	–	2
Colon (2)	2	–
Soft tissue (2)	1	1
Omentum (1)	–	1
Total (18)	8	10

The 10 true negative samples for EPTB, were negative by all tested methods including cloning and were used to calculate the specificity of the applied methods. Table 3 represents true positive and true negative samples detected by each PCR method and sensitivity, specificity and 95% CI in relation to the histopathology results.

**TABLE 3:** **True positive and true negative samples detected by each method and sensitivity, specificity and 95% confidence intervals in relation to the histopathology results**.

**Methods**	**True positive No. (%)**	**Sensitivity**	**95% confidence interval**	**True negative No. (%)**	**Specificity**	**95% confidence interval**
*rpoB*- PCR	14/70 (20)	20%	(11.39–31.27%)	10/10 (100)	100%	(69.15–100%)
*IS6110*- PCR	34/70 (48.6)	48.6%	(36.44–60.83%)	10/10(100)	100%	(69.15–100%)
*rpoB*-nested PCR	60/70 (85.7)	85.7%	(75.29–92.93%)	10/10(100)	100%	(69.15–100%)
Nested PCR–cloning	67/70 (95.7)	95.7%	(87.98–99.11%)	10/10(100)	100%	(69.15–100%)

## Discussion

Extra pulmonary tuberculosis is a significant health problem in both developed and developing countries. Literature reviews reveal that it accounts almost one of the three of total cases of TB in the world. In a study enrolled in US on 253229 cases of TB during 14 years, more than 18.7 percent were diagnosed for EPTB (Peto et al., 2009). Moreover, WHO statistical data shows that TB is the main cause of 66,000 death in year which is equivalent to eight death per hour in European countries. In Iran, EPTB is accounts for more than 29 percent of all TB cases (World Health Organization, 2013). Therefore, EPTB cases constitute a large number of TB burden which needs serious attentions of public health authorities in diagnosis and their identification in order to treat them properly.

The major problem is that the diagnosis of the disease in its different clinical presentations, remains a true challenge (Cailhol et al., 2005). The lack of a sensitive, specific and rapid method for the early diagnosis of EPTB poses a difficulty in initiating early therapy (Ong et al., 2004). Fresh clinical samples are desired for laboratory diagnosis of mycobacterial infections. However, in most cases of EPTB, fresh samples are not possible to obtain, so, in such cases, formalin-fixed paraffin-embedded tissue samples are used. These samples comprise technical limitations, including the lack of ability to culture. So the histopathology screening and PCR methods are the valuable diagnostic tools. For PCR assay, choosing one or more appropriate target (s), can be a great way to achieve a high sensitivity. PCR targeting *rpoB*, IS*6110* genes and nested PCR have been generally used to detect MTBC in EP samples (Yun et al., 2005; Huang et al., 2009; Maurya et al., 2012).

In present study from 70 samples have been confirmed by histopathology, 20, 48.6, and 85.7% were positive by *rpoB-*PCR, IS*6110*-PCR and nested- *rpoB* PCR respectively, and 10 samples (25.7%) were either weak positive or negative with nested- *rpoB* PCR method. In study of Chakravorty et al. (2005), 99 EP specimens were screened for the detection of MTB by PCR targeting *devR* and IS*6110* genes and their results showed positivity rates of 66.7 and 83% respectively, and 87.5% for the combination of both assays. In comparison, our findings revealed lower positivity rate by using IS*6110* -PCR. In study of Hillemann et al. (2006), 24 paraffin-embedded tissue specimens were studied for the presence of MTBC and they concluded that the real-time PCR assay exhibits a higher sensitivity (66.7%) for the detection of MTBC DNA compared to an alternative in-house IS*6110* PCR (33.3%). As shown in their study, the rate achieved by IS*6110*- PCR was lower than our results. IS*6110* is one of the main targets for the detection of MTB isolates, however, in this study, in comparison to nested *rpoB* PCR, the rate of positivity was lower. This positivity rate difference, may be due to chemical and physical alterations on DNA during fixation and tissue preparation (Barcelos et al., 2008), or is a 0-band strain (Lok et al., 2002).

**FIGURE 3:**
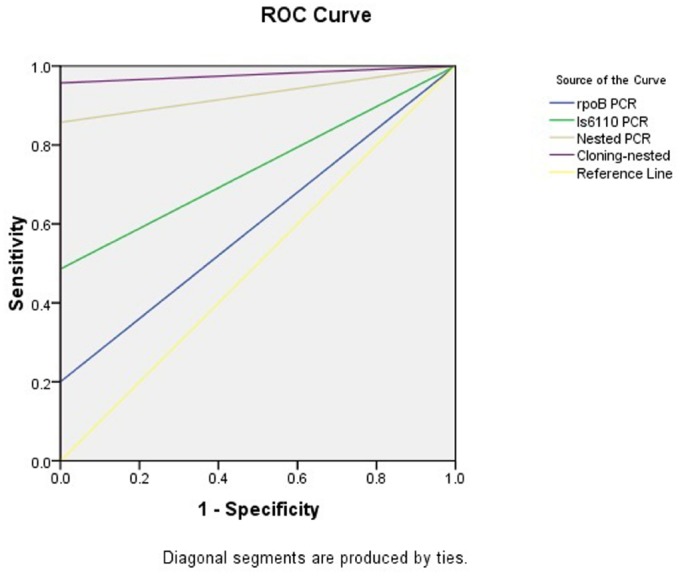
**The area and the sensitivity and specificity of ROC curves of the methods applied in present study**.

The *rpoB*-PCR alone showed low sensitivity in this study, but when this target gene was used in nested PCR, we had a high sensitivity of 85.7%. This was in concordance with similar study of Huang et al. (2009) which a rate of 86.1% was reported. By using TA cloning and subsequent sequencing of the cloned plasmid, we gained an overall 95.7% sensitivity compared to histopathology examination leading to construct a ROC curve analysis (Figure 3), which the PCR methods results variable related to curve are presented in Table 4. The nucleic acid sequence analysis revealed a 98–100% homology in comparison with MTB H37Rv reference strain (Genbank). TA cloning showed much higher sensitivity compared to other applied techniques. By cloning, we were able to first, detect seven additional positive samples among the initial 10 negative samples in the PCR methods including nested *rpoB*-PCR and second, improve the quality of eight samples with weak positive results by nested *rpoB*- PCR, allowing its sequencing.

**TABLE 4:** **The test results variable(s): *rpoB* PCR, IS*6110* PCR, Nested PCR, Cloning-nested PCR**.

**Test result**				**Asymptotic 95% confidence interval**	
**variable(s)**	**Area**	**Standard error^a^**	**Asymptotic Significance^b^**	**Lower bound**	**Upper bound**
*rpoB* PCR	0.600	0.083	0.309	0.436	0.764
IS*6110* PCR	0.743	0.062	0.013	0.622	0.864
Nested PCR	0.929	0.028	0.000	0.873	0.984
Cloning-nested PCR	0.979	0.015	0.000	0.949	1.008

The PCR methods has at least one tie between the positive actual state group and the negative actual state group. Statistics may be biased.

^a^Under the non-parametric assumption.

^b^Null hypothesis: true area = 0.5.

However, despite the advantages of TA cloning, it is a costly and not easy to perform technique and is not applicable for routine use in microbiology laboratories.

There were also some limitations in our study. Unfortunately, we had no access to the fresh or frozen samples and all the specimens were archived FFPE tissues. In such samples, the DNA integrity could vary and the degradation might be increased. In study of Ritis et al. (2000), the nested PCR and conventional methods for the diagnosis of EPTB samples were compared. According to their findings, the positivity rate by nested PCR was 90.9%, while conventional Acid fast microscopy and culture methods showed only 18.2% positivity. The samples in their study were fresh EP samples, which comprise less difficulties in processing and expecting to achieve more sensitivities compared to FFPE tissue samples. However, the results from combination PCR-TA cloning methods in our study, were in concordance to the results of histopathology examination and made us sure about the true positive rate despite the nature of our tested samples. For a country surrounded with very high incidence and prevalence of TB such as Afghanistan, Pakistan, Azerbaijan, Tajikistan and Iraq, there is an urgent demand to settle down a guideline for appropriate molecular diagnostic methods with high sensitivity and specificity. Khuzestan province is in close contact with Iraqi population which needs more collaborative public health measurement by the authorities in the south-western region of Iran.

Our results indicated that from three PCR assays applied on samples, nested- *rpoB* PCR showed a high detection rate for MTBC DNA. Due to several problems are associated with the detection and identification of MTBC in FFPE tissues, nested-*rpoB* PCR combined with TA cloning-sequencing can be a useful method for the detection of MTBC DNA in samples from EPTB. In this study we gained a true positive rate of 95.7% by this combination compared to histopathology examination (70 samples) with 95% CI for our estimates of sensitivity and specificity. Further investigation with larger number of samples specially fresh biopsies and using more molecular targets are urge to perform in order to evaluate our results.

### Conflict of Interest Statement

The authors declare that the research was conducted in the absence of any commercial or financial relationships that could be construed as a potential conflict of interest.
